# Comprehensive analysis of transglutaminase substrate preference by cDNA display coupled with next-generation sequencing and bioinformatics

**DOI:** 10.1038/s41598-022-17494-4

**Published:** 2022-08-09

**Authors:** Jasmina Damnjanović, Nana Odake, Jicheng Fan, Maurizio Camagna, Beixi Jia, Takaaki Kojima, Naoto Nemoto, Kiyotaka Hitomi, Hideo Nakano

**Affiliations:** 1 Laboratory of Molecular Biotechnology, Graduate School of Bioagricultural Sciences, Nagoya University, Furo-cho, Chikusa-ku, Nagoya, 464-8601 Japan; 2 Laboratory of Plant Genetics and Breeding, Graduate School of Bioagricultural Sciences, Nagoya University, Furo-cho, Chikusa-ku, Nagoya, 464-8601 Japan; 3 Laboratory of Evolutionary Molecular Engineering, Graduate School of Science and Engineering, Saitama University, 255 Shimo-Okubo, Sakura-ku, Saitama, 338-8570 Japan; 4 Laboratory of Cellular Biochemistry, Graduate School of Pharmaceutical Sciences, Nagoya University, Furo-cho, Chikusa-ku, Nagoya, 464-8601 Japan; 5 Present Address: Tigermed, Hangzhou, China; 6 Present Address: Faculty of Agriculture, Meijo University, 1-501 Shiogamaguchi, Tempaku-ku, Nagoya, 468-8502 Japan

**Keywords:** Biochemistry, Biocatalysis, Protein design, Biotechnology, Next-generation sequencing

## Abstract

cDNA display is an in vitro display technology based on a covalent linkage between a protein and its corresponding mRNA/cDNA, widely used for the selection of proteins and peptides from large libraries (10^12^) in a high throughput manner, based on their binding affinity. Here, we developed a platform using cDNA display and next-generation sequencing (NGS) for rapid and comprehensive substrate profiling of transglutaminase 2 (TG2), an enzyme crosslinking glutamine and lysine residues in proteins. After screening and selection of the control peptide library randomized at the reactive glutamine, a combinatorial library of displayed peptides randomized at positions − 1, + 1, + 2, and + 3 from the reactive glutamine was screened followed by NGS and bioinformatic analysis, which indicated a strong preference of TG2 towards peptides with glutamine at position − 1 (Gln-Gln motif), and isoleucine or valine at position + 3. The highly enriched peptides indeed contained the indicated sequence and showed a higher reactivity as TG2 substrates than the peptide previously selected by phage display, thus representing the novel candidate peptide probes for TG2 research. Furthermore, the obtained information on substrate profiling can be used to identify potential TG2 protein targets. This platform will be further used for the substrate profiling of other TG isozymes, as well as for the selection and evolution of larger biomolecules.

## Introduction

Transglutaminases (TGs: EC 2.3.2.13) are enzymes catalyzing transamidation, a transfer reaction between an acyl donor (peptidyl glutamine) and an acyl acceptor (amino group of lysine). As a result, a stable isopeptide bond is formed, resistant to proteolytic degradation. Owing to their transamidation activity, TGs are known for their role in crosslinking of proteins and peptides. Since transamidation proceeds via the formation of an acyl-enzyme intermediate, which is a rate-limiting step, TGs show high specificity towards acyl donors, while reacting on a variety of acyl acceptors such as lysine residues of proteins and small primary amines^1^.

The presence of TGs is vast across kingdoms of plants, animals and microorganisms. Microbial TGs have been involved in a variety of biotechnological applications and industrial use^2–4^. In mammals, eight different types of TGs have been identified (TG1, TG2, TG3, TG4, TG5, TG6, TG7, and factor XIII), with functions ranging from blood clotting, epidermis and hair follicle formation, wound healing, apoptosis, extracellular matrix formation and cell adhesion^5^. Aberrant expression and function of TGs cause serious effects on human health and cause conditions such as hemorrhage, celiac disease, cancer, fibrosis, Alzheimer's and Huntington’s disease, and lamellar ichthyosis^6^. Although widely studied, more work is needed to fully understand the biological function of TGs, for which sensitive and specific in situ detection of TG activity is desired. Artificial fluorescently labeled Gln-peptide probes (‘Hitomi peptides’) have greatly contributed to in vitro and in situ detection and measurement of TG activity^7–12^. These probes have been developed by selection from random peptide libraries using phage display technology, and are available for studies of TG isozymes. However, to this date, these probes have not been optimized in terms of amino acid preference at Gln-surrounding positions or used to investigate TG substrate preference in detail.

An in vitro screening method, mRNA/cDNA display, pioneered by two groups, Roberts and Szostak’s group^13^ and Nemoto and Yanagawa’s group^14^ relies on the formation of a covalent link between the genotype (mRNA or cDNA) and phenotype (protein of interest) via puromycin. The convenience of complete control of expression/screening conditions, stable genotype–phenotype linkage, incorporation of unnatural amino acids and ability to handle large libraries (up to the 10^12^ variants) makes mRNA/cDNA display a preferred screening method.

Numerous reports exist on mRNA/cDNA display being applied to affinity-based screening and selection of peptides and antibody fragments^15–21^. Utilization of mRNA/cDNA display for activity-based selection of enzyme substrates has been described to a much lower extent. The representative applications include substrate profiling of protein-modifying enzymes in proteomics research, such as caspase^22^, viral protease and a kinase^23^, metalloprotease^24^, and enzymes involved in the modification of post-translationally modified peptides^25,26^, and demonstrate that substrate preference can be studied in great detail by mRNA/cDNA display. Aiming to study the substrate preference of microbial transglutaminase (MTG) and design its artificial substrates for biotechnological applications by mRNA display, Lee et al. reported on isolating a specific and reactive Gln probe for MTG after six rounds of screening and selection from a 10-mer random library^27^.

**Figure 1:**
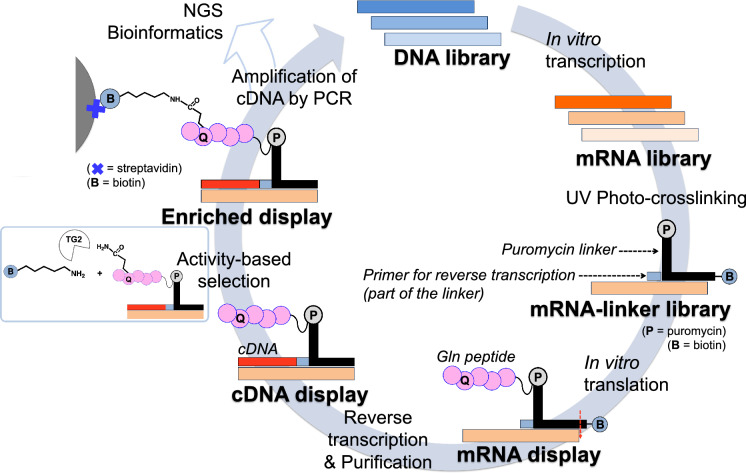
Outline of the cDNA display platform for screening and selection of preferred TG2 peptide substrate. TG2 stands for transglutaminase 2. Red dotted line represents cleavage site of RNase T1.

Here, we established a cDNA display/next-generation sequencing (NGS) platform for comprehensive analysis of the TG substrate preference (Fig. 1), which encompasses the majority of reactive sequences rather than just the best hits, and allows us to understand the importance of selected amino acid residues at each position of the peptide and their properties for the enrichment. We also aimed to reduce the time and labor associated with multiple selection rounds and probe the sensitivity of the platform in a single selection round. As a target TG, we chose mammalian TG2 isozyme for its omnipresence in cellular compartments and tissues and its importance in many cellular processes^28,29^. Its artificial substrate, T26 peptide, previously isolated from a phage-displayed random 12-mer-peptide library^11^, was used as a template for peptide library generation.

## Results

### Preparation of displayed libraries

To optimize the selection and test its efficiency, we first prepared binary model libraries consisting of T26 peptide and non-substrate peptide, T26A (with Q2A mutation), by mixing corresponding dsDNA in specified molar ratios. Prepared DNA pools were used as templates for mRNA library generation as described in Methods (Fig. S1B). mRNA synthesis on a 30-μL scale proceeded with an overall yield of 29 μg for the 1:4 mRNA model library and 55 μg for the 1:50 mRNA model library. Random library LibQ consisted of peptide sequences with T26 backbone randomized at the reactive Gln and was used as a control to verify the appropriateness of the selection, and data acquisition and analysis. Lib4 consisted of peptide sequences with T26 backbone randomized at positions of interest around the reactive Gln, namely − 1, + 1, + 2, and + 3, suspected to be important for the reactivity of the peptide in the TG2 reaction. Random dsDNA libraries were prepared from chemically synthesized ssDNA and converted into the corresponding mRNA libraries as described in Methods. mRNA synthesis at a 30-μL scale proceeded with the overall yield of 36 μg for the Lib4 mRNA library and 56 μg for the LibQ mRNA library. The formation of mRNA-linker complexes was confirmed for all libraries (left panels of Fig. S1B and C). Obtained mRNA-linker libraries were used as templates for in vitro translation to obtain mRNA display libraries (right panels of Fig. S1B and C).

### Selection of T26 sequence from binary model libraries

The selection step relies on TG2-mediated crosslinking of displayed Gln peptides with biotin-pentylamine resulting in biotinylation of the reactive peptide sequences which can be captured by streptavidin-coated magnetic microbeads and pulled down. After the selection and subsequent processing, the cDNA of the original (display pool before selection), enriched (display pool enriched on the surface of the microbeads), and leftover (display pool remaining in the solution after selection) pools were PCR-amplified by step 1 of the nested PCR, digested with *Sph*I and analyzed by agarose gel electrophoresis (Fig. S2A). Band density analysis indicates enrichment factors (EF) of 5 and 30 for 1:4 and 1:50 libraries respectively.

### Recovery of DNA after selection from random libraries

The selection and subsequent processing proceeded as outlined in Methods. cDNA of the original, enriched and leftover pools was PCR-amplified by step 1 of the nested PCR, and analyzed by agarose gel electrophoresis. Bands corresponding to the original and leftover DNA pools were observed (Fig. S2B). Since the corresponding bands were not observed for the enriched DNA pools, step 2 of the nested PCR was performed with different volumes of the PCR reaction mixture from step 1 as a template. After step 2, bands corresponding to the DNA of the enriched DNA pools were detected (Fig. S2C). We believe that due to the low amount of the reactive peptide sequences, an initial amount of cDNA enriched on the beads was too low to obtain a detectable amplification product after a single round of PCR. The amplified DNA of the original LibQ and Lib4 libraries and enriched LibQ and Lib4 libraries (fraction S1 of the 2nd Nested PCR) were purified and prepared for NGS analysis.

### Analysis of the sequencing data from random libraries

**Figure 2:**
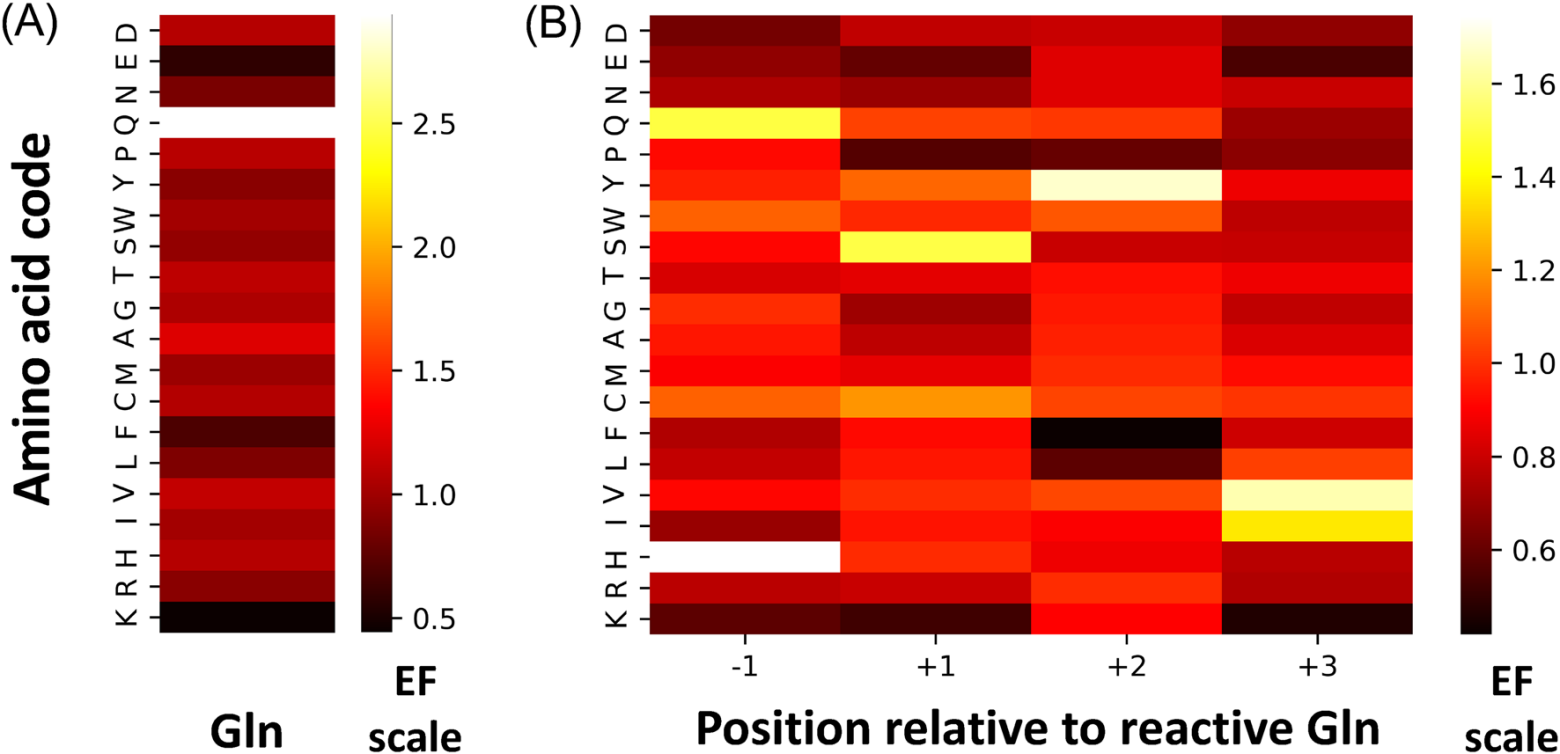
The heatmaps showing the color-coded enrichment factor of each amino acid at randomized positions of (**A**) LibQ and (**B**) Lib4.

The raw NGS data were analyzed as described in Methods. EFs of amino acids at each randomized position were calculated and analyzed to understand amino acid sequence enrichment by position. The EF of Gln from LibQ was approx. 3 (Fig. 2A).

From Lib4, His and Gln were predominantly enriched at position − 1, Ser and Cys at position + 1, Tyr at position + 2, and Val and Ile at position + 3 (Fig. 2B). Based on the analysis of the position-specific sequence preference two common sequences can be observed, the original T26 sequence (H-Q-S-Y-V-) and the heatmap-derived sequence Q-Q-C-Y-I-.

The list of top 100 peptide sequences enriched from Lib4 and ranked by the EF is given in Table S1. The sequence logo of the top 100 peptides is shown in Fig. S3. Among the top 100 enriched peptides, approx. 30%, including the first six sequences, contain the Gln-Gln motif in positions − 1 and 0, in accordance with the heatmap-derived sequence. In a few sequences, the Gln-Gln motif is present at 0 and + 1 positions. Interestingly, the selected sequences also show the presence of Cys, mostly at positions − 1 and + 1, around the reactive Gln. In the top 100 sequences, position + 1 is mostly occupied by Tyr (23%) followed by Cys, Val and Phe (12 and 14% for Phe). Position + 2 shows less preference for any particular residue, while at position + 3, Val and Ile are dominant. The original T26 peptide was also enriched from Lib4 and ranked 2466th with an EF of approx. 2. For comparison, the most enriched peptide, Top 1 (Table S1) had an EF of approx. 7.

### Reactivity of the selected peptides in enzymatic TG2 assay

**Figure 3:**
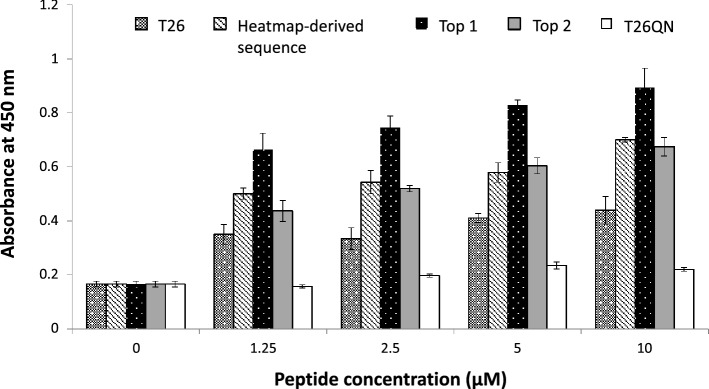
Reactivity of the top enriched peptides, Top 1 and Top 2, and heatmap-derived sequence (Q-Q-C-Y-I-) in comparison to T26 and non-substrate peptide, T26QN, determined by the TG2 enzymatic assay. Assay was carried out in plate format using biotinylated peptides in the concentration range 0–10 μM with immobilized casein as amino donor, as described in Methods. Reaction products were detected by absorbance at 450 nm. Bars represent means of triplicate measurements with STD indicated for each measurement. All peptides were chemically synthesized with N-terminal biotin tag by Bio-Synthesis, USA and provided by Biologica, Japan.

The two peptides with the highest EF, Top 1 and Top 2 (Table S1), T26, a peptide with heatmap-derived sequence and T26QN (a non-substrate peptide with Q2N mutation) were used for the evaluation of their reactivity in TG2 enzymatic plate assay. Results indicate that all but the T26QN peptide showed reactivity with TG2 (Fig. 3). The reactivity of the Gln-containing peptides increased proportionally to their concentration. The Top 1 peptide had the highest reactivity, while both the Top 2 and the heatmap-derived sequence peptide showed a similar, but slightly lower reactivity than Top 1. Both newly selected peptides showed higher reactivity than the T26 peptide, previously selected by phage display and widely used in many research areas.

### Importance of selected amino acid residues and their properties for enrichment

**Figure 4:**
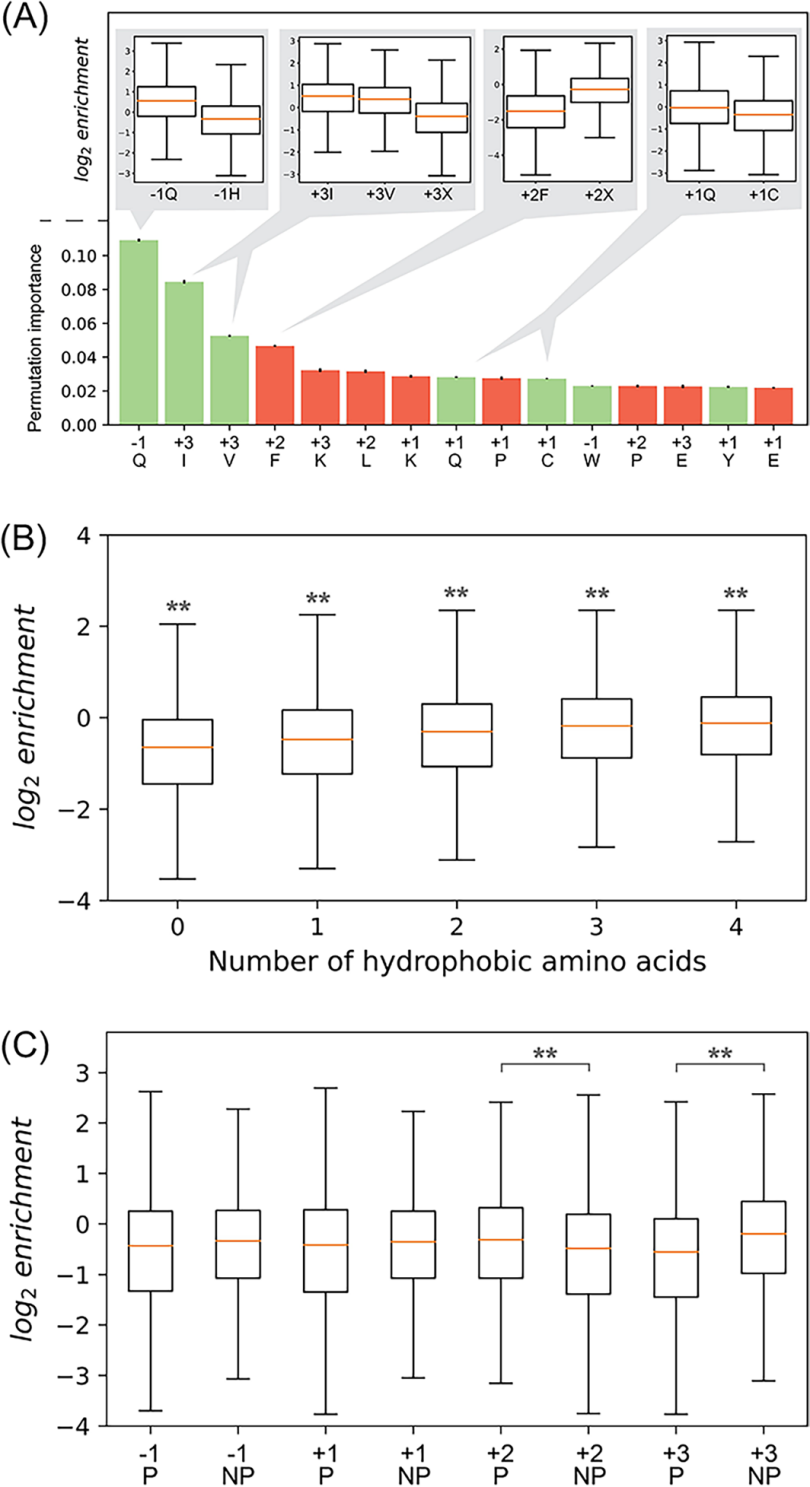
Analysis of the enriched peptides from Lib4. (**A**) Permutation importance for the amino acids used as input for a Random Forest Regression model, sorted by importance. The permutation importance indicates how important given amino acid is to accurately predict enrichment. Color of bars indicates association with enrichment: Green = important for increase of enrichment, red = important for decrease of enrichment. Bars are mean permutation importance for ten-fold cross-validated calculations. Insets represent enrichment differences at positions − 1, + 1, + 2 and + 3 when a particular amino acid is in that position (left side) and when any other amino acid is in that position (right side), and are significant as determined by independent T-test (*p* < 0.01; n > 7000). X denotes any amino acid except the one analyzed in the bar/s to the left in each inset. (**B**) Effect of hydrophobicity on enrichment. Asterisks indicate significant difference to all other plots, determined by independent T-test (*p* < 0.01; n = 7988–35,482). (**C**) Effect of polarity on enrichment. Amino acids with polar residues are denoted with P, and non-polar with NP. Asterisks indicate significant difference, determined by independent T-test (*p* < 0.01; n > 70.000). Orange lines in all boxplots indicate median enrichment; boxes represent first and third quantile; whiskers represent 1.5 × inter-quantile range.

We used a supervised learning algorithm, Random Forest, to analyze the enriched peptide data set of Lib4 and study the importance of amino acid residues and their properties for peptide enrichment. The results are summarized in Fig. 4. For the increase of enrichment, the highest importance is assigned to Gln at position − 1, followed by Ile/Val at position 3 and Gln/Cys at position 1 (Fig. 4A) indicating these sequences as the most preferred by TG2. For the decrease of enrichment, the highest importance is assigned to Phe at position 2 followed by Lys at position 3, Leu at position + 2 and Lys/Pro at position + 1, indicating the sequences disliked by TG2. The insets of Fig. 4A show the enrichment changes when the residues of high importance at positions − 1, + 1, and + 2 are replaced by other indicated residues. It should also be noted that the polarity of the residues and overall hydrophobicity of the randomized region also has an impact on the enrichment. We found that an increasing number of hydrophobic amino acids had a positive effect on enrichment (Fig. 4B). The effect of hydrophobicity is relatively weak, seen that most of the top 100 enriched peptides only contain three hydrophobic amino acids, and the impact of some highly beneficial or detrimental amino acids at certain positions takes precedence over hydrophobicity. Nonetheless, hydrophobicity does have an impact on enrichment and this effect may eventually be leveraged for protein engineering purposes. Similarly, we found that the polarity of amino acid residues had a position-dependent effect. While polarity at positions − 1 and + 1 does not have a significant impact on enrichment, it appears to have a significant effect at positions + 2 and + 3. A polar residue at + 2 is associated with an increase of enrichment, whereas the opposite is observed at position + 3 (Fig. 4C).

### Identification of new potential protein targets of TG2

We used NCBI Blastp (https://blast.ncbi.nlm.nih.gov/Blast.cgi) search with the first seven residues of the enriched sequences (Top 1, Top 2), T26 peptide and heatmap-derived sequence as a query against a non-redundant sequence database of mouse and human proteins to verify if potential new protein targets of TG2 can be identified. Based on the search results summarized in Table 1, various proteins with diverse functions contain sequences with matching motifs. The localization of these potential target proteins is consistent with that of TG2. As an unexpected finding, the searches converge towards variable and junction regions of immunoglobulins, indicating a possible role of TG2 in the regulation of antibody function.

**Table 1:** Summarized results of the Blastp search with four query sequences, consisting of the first 7 a.a. residues of the peptides against the human and mouse proteins.

Query	T26 (HQSYVDP)	Heatmap-derived sequence (QQCYIDP)
Match	Ig light chain junction region Ig heavy chain junction region Ig kappa light chain variable region Ankyrin-3 isoforms mCG144990 Histone-lysine N-methyltransferase SETD2 isoforms Bombesin receptor Synaptogyrin-2 hCG1820523	Zinc transporters (solute carrier 30 family) Ig kappa light chain variable region Ig light chain junction region Heparanase 2 and 3 Coatomer subunit beta Telomere length regulation protein TEL2 Gamma-glutamyl hydrolase precursor mCG12390 IgA heavy chain variable region
**Query**	**Top 1 (QQVCIDP)**	**Top 2 (QQYVVDP)**
Match	Stromal cell-derived factor 1 Fanconi anemia group A protein Ig heavy chain junction region Signal peptide, CUB and EGF-like domain-containing protein 2 mCG4615 Testase 1 FERM and PDZ domain-containing protein 1 Amiloride-sensitive sodium channel subunit alpha Olfactory receptors Seven-transmembrane helix receptor Protein C9orf135 isoform X2 Deleted in lung and esophageal cancer protein 1 C-X-C motif chemokine 6 precursor Granulocyte chemotactic protein mCG147083	Anaphase-promoting complex subunit 1 Ig light chain junction region Chromosome 8 ORF 53 (human) mCG1045346 Vitrin isoforms Ig heavy chain junction region Triple functional domain protein Kalirin

Only the matches with 6–7 residues including one residue mismatch, or 5 residues without a mismatch are given. If aligned amino acid residues are not identical but are similar in size and nature, this was not considered a mismatch.

## Discussion

The platform for rapid selection and comprehensive analysis of TG substrate preference has been established and used to analyze the substrate profile of TG2. This in vitro system enables rapid selection of preferred TG substrate peptides from random libraries, resulting in enrichment of the selected sequences on the surface of the magnetic microbeads, from where they can be recovered and analyzed. We combined cDNA display technology with NGS to yield a system for the rapid activity-based evolution of peptides from large libraries in the shortest time with minimal demand for labor and, with full control over the expression conditions. The use of NGS increases the throughput and enables comprehensive analysis since we can gather broad but accurate information on the enriched sequences. Accuracy of the NGS can shorten the selection to only one or a few rounds, based on which we can deduce the substrate preference by proper data processing and analysis.

T26-derived peptide libraries were displayed with high translation efficiency and high solubility of the mRNA display complex (Fig. S1). It is believed that the formation of the mRNA display complex itself helps the peptide stay soluble and available for the enzymatic reaction.

Analysis of DNA pulled down from LibQ indicated that Gln was predominantly enriched (Fig. 2A). The lower-than-expected enrichment factor for Gln may have been caused by non-specifically enriched peptide sequences, either by direct binding to the surface of the beads or the beads-immobilized streptavidin. However, Gln was the only enriched sequence (EF > 1).

In Lib4, we first considered position-specific sequence preference (Fig. 2B). Preference for Gln at position − 1, next to the reactive Gln, is well aligned with other studies reporting the Gln-Gln and Gln-Gln-Gln motif in the preferred sequences of TG2^30^ and microbial TG^27^. These motifs are also present in natural protein targets of TG2, such as substance P, crystallin, and fibronectin, as well as α-gliadin, one of the gluten peptides. Negatively charged Asp and Glu, as well as Lys, were the least preferred residues at position − 1. Lys is expected to be among the least preferred residues due to its reactivity as an acyl acceptor of the neighboring Gln, which could lead to self-cross-linking of the peptide.

At position + 1, the most preferred residues, Ser and Cys, are similarly shaped, small polar amino acids. Interestingly, the presence of Cys in the peptide sequence did not prove harmful to the enzyme’s active site Cys, possibly due to the reducing environment of the selection reaction. The least preferred residues at this position were Pro and Glu as well as Lys, likely due to its reactivity leading to the peptide self-cross-linking.

Besides considering position-specific sequence preference, we have also analyzed the top 100 enriched peptide sequences ranked by the enrichment factor (Table S1). The sequences are well aligned with the heatmap-derived sequence, however with a few differences. Instead of His and Gln, the top peptides show predominantly Gln at position − 1 (28%). The abundance of Tyr at position + 1 (23%) and the rather broad presence of hydrophobic and basic residues at position + 2 are also evident. Position + 3 is dominated by Val and Ile, as also observed in the heatmap-derived sequence. It should be noted that before the last filtering to remove peptides without the reactive Gln, the top 100 enriched peptides list contained seven sequences without Gln. Their presence in the original library could be explained by errors in the chemical synthesis of ssDNA library fragments. Upon inspection of their sequences, we noticed that these peptides contain His and Trp near the N-terminus, which is a characteristic of streptavidin-binding peptides, implying that these sequences could have been bound to streptavidin and falsely enriched during the selection, as described before^11^. Another unexpected finding in the top 100 enriched peptides list is the presence of six peptides with backbone sequences (non-randomized part of the peptide) different from the template T26 peptide. We suspect that these sequences originate either from random errors in the initial ssDNA synthesis or erroneous base calls during the NGS. Although the latter seems less likely due to the good data quality and general reliability of Illumina sequencing. Since these peptides had Gln at the expected position and could become TG2 substrates, we considered their ranking and EF.

The results of the TG2 in vitro assay undoubtedly show that the selected peptides are indeed appropriate substrates of TG2, with higher reactivity than the original T26 peptide (Fig. 3). Thus, we conclude that the enriched peptides represent good candidates for the development of highly sensitive TG2 peptide probes for in situ TG2 detection. In the future, we plan to evaluate the newly selected peptides in more detail to understand whether they are specific TG2 substrates or they show reactivity towards other TG isozymes.

As expected, results of the Random Forest analysis of the current data set suggest that the highest importance beneficial for enrichment is the presence of Gln at position − 1 (Fig. 4A). This prediction is well aligned with the heatmap-derived sequence and the sequences of the top 100 enriched peptide group, and it is further supported by the high reactivity of the peptides with this sequence in the TG2 enzymatic assay. High importance beneficial for enrichment is also assigned to Ile and Val at position + 3, which is consistent with the heatmap-derived sequence and top 100 enriched peptide group. For position + 1, the prediction of importance points at Gln, Cys and Tyr as beneficial for enrichment, while the heatmap-derived sequence indicates Ser and Cys, and the top 100 enriched peptide group indicates Tyr. From all data, we conclude that all four suggested residues have some positive impact on reactivity but not as much as specific sequences at positions − 1 and + 3. Similarly, position + 2 seems to tolerate diverse sequences, including hydrophobic ones except for Phe, Leu and Pro, as also observed in the heatmap-derived sequence. The 100 top enriched peptide group, besides hydrophobic residues, at position + 2 also shows the significant presence of basic residues, which are predicted by Random Forest to have a positive impact on the enrichment (Fig. 4C).

It is important to mention that the top enriched peptide sequences identified in this work are largely in agreement with those selected in previous works investigating TG2 substrate preference by different technologies and using different library design. Sugimura et al.^11^ and Keresztessy et al.^30^ used phage-displayed random peptide libraries to select preferred TG2 Gln peptide sequences. A number of isolated sequences in this and both previous studies have Gln-Gln motif at or near the N-terminus, while position + 3 includes aliphatic amino acids namely, Val, Leu, Ile, as well as Met. The sequence at these two positions indeed proved to have the highest importance in our study. A notable difference between this and the previous two studies is the abundance of Pro at position + 2, reported by Sugimura et al. and Keresztessy et al. however not observed in our data set. One possible reason is a different library design, where our Lib4 library had less diversity and a common backbone unlike the other two. In all three studies, position + 1 showed greater variation and sequence tolerance. Another study on substrate preference of TG2 used database search and regression analysis to predict the possible TG2 substrate sequences which were later tested^31^. The authors found that Gln and Gly at position − 1, Gln at position + 1, and Gln and Lys at position + 2 had a positive impact on peptide reactivity with TG2. Furthermore, Csosz et al. reported on the negative impact of Leu at position − 1 and Ser at position + 2 on reactivity as well as predicted the spatial proximity of His, Val, and to some extent, Arg, to have the highest positive impact on the reactivity while the proximity of Asp is predicted to have the highest negative impact. Our study could confirm the positive effect of Gln at positions − 1, + 1, and + 2, although their importance greatly varied. While the most important proved to be Gln at − 1, Gln at + 1 and + 2 was of much less importance. Similarly, Gly at − 1 had and Lys at + 2 showed a positive impact on reactivity however of low importance.

Upon inspection of the TRANSDAB database (http://genomics.dote.hu/mediawiki/index.php/Main_Page), and while considering only the proteins with known TG2 substrate sequences, we could observe significant similarities between our top enriched peptide sequences and substrate sequences determined to be natural targets of TG2. A great number of sequences contain the Gln-Gln motif, and an even greater number shows Val or Ile at position + 3 (Table 2). We could also observe the presence of hydrophobic and basic residues at position + 2, while at position + 1 we could not identify any specific sequence or type of amino acid. Interestingly, insulin, also identified as a TG2 target, includes a substrate motif rich in Cys at positions + 1 and + 2 or + 3. Our study also identified enrichment of Cys at positions + 1 and + 2.

**Table 2:**
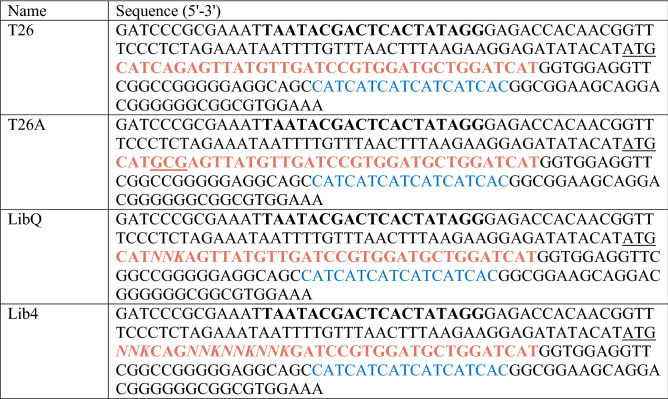
DNA sequences of the cDNA display constructs used in this study.

T7P sequence is bolded, peptide sequence is colored red, and 6xHis sequence blue. Randomized sites are italicized.

Preferred peptide sequences of other TG isozymes identified by phage display^7,10,12,32^ show low resemblance to our top enriched peptides. Notable similarities include the preference for hydrophobic residues at position + 3 in peptide substrates of TG1, TG3, TG6, and TG7. In addition, peptide substrates of TG1 and TG3 show a preference for Arg and Lys at position + 2. Because of the low sequence similarity with preferred substrates of other TG isozymes, we expect that the peptides isolated in this study are TG2-specific Gln peptide substrates.

Finally, the results of our Blastp search (Table 1) suggest that our platform could be used for the identification of possible novel TG2 targets. Immunoglobulins (Igs) are suggested as targets in all queries and represent the first candidates for further testing against TG2. The role of TG2 in Ig modification has been scientifically described in the work of Iversen et al.^33^ where the authors demonstrated that Igs, in particular IgD, can be substrates of TG2 and suggested that TG2-mediated crosslinking occurs either between two Igs or between Ig and gluten peptide, although the exact TG2 substrate sequence has not been identified.

Although we selected glutamine peptide sequences using biotinylated pentylamine as an acyl acceptor, which is a different substrate from the natural protein acyl acceptors, we believe that the results of our screening can be taken as a general guide towards the choice of proteins to be further investigated by classic proteomic methods as natural TG2 targets.

This is the first report on rapid and comprehensive TG profiling using the molecular display. This platform can be applied to screen for the substrate preference of other mammalian TGs and design appropriate peptide probes for the specific detection and analysis of TG activity in tissue samples. In continuation, the platform is expected to become a valuable tool for the selection of biomacromolecules such as proteins during directed evolution.

## Methods

### Materials

Oligonucleotides were synthesized either by Greiner Bio-One, Integrated DNA Technologies or by Eurofins. Restriction enzymes, DNA polymerases, Klenow fragment and Recombinant RNase Inhibitor were purchased from Takara Bio. Recombinant Transglutaminase 2 was from Novus Biologicals, USA. Components of in vitro cell-free protein synthesis kit, PURE*frex*, were provided by GeneFrontier, Japan. Puromycin cnvK linker was obtained from Epsilon Molecular Engineering Inc., Japan. Pentylamine-biotin, RNase T1, SYBRGold, and streptavidin-coated magnetic beads Streptavidin MyOne C1 were purchased from Thermo Fisher Scientific. N-terminal biotinylated peptides Top 1, Top 2, T26, T26QN and peptide with heatmap-derived sequence were chemically synthesized by Bio-Synthesis, USA and provided by Biologica, Japan.

### Preparation of DNA constructs

Plasmids with the T26 gene and its corresponding non-substrate version, T26A with Q2A mutation were prepared as described in the *Supplementary material* (Methods section).

### Preparation of DNA templates for cDNA display

DNA sequences of the cDNA display constructs used in this study are given in Table 2.

Preparation of the DNA templates for the display of binary model libraries is described in the *Supplementary material* (Methods section; Fig. S1A). For the preparation of random libraries, LibQ and Lib4, 61-basepair peptide gene ssDNA consisting of Gln replaced by degenerate NNK codon, and amino acid sequences at positions − 1, + 1, + 2, + 3 from Gln replaced by degenerate NNK codon respectively, were custom ordered from Integrated DNA Technologies. The scheme of library preparation is given in Fig. S4. dsDNA fragments were generated in the reaction with Klenow fragment and a single primer, number 7 (Table S2), and inserted into PCR-amplified (primers 8–9 in Table S2) and purified pRSET vector fragment by Gibson Assembly (NEB) to add sequence parts necessary for cDNA display generation to the DNA library. Assembled products were PCR amplified using New Left and cnvK_New Ytag primers, column-purified and used for in vitro transcription to obtain LibQ and Lib4 mRNA libraries.

### Preparation of cDNA display

mRNA pools were prepared by in vitro transcription using the RiboMAX Large Scale RNA Production System-T7 (Promega) and prepared DNA templates. For binary model libraries, DNA of T26 and T26A sequences were mixed in designated molar ratios and used as templates for the synthesis of the mRNA library. For random libraries, prepared dsDNA of LibQ and Lib4 were used as the templates. The reaction mixtures were incubated at 37 °C for 2 h. This was followed by purification of the synthesized mRNA, including the on-column DNA digestion, with the NucleoSpin RNA kit (Takara Bio). The purity and concentration of RNA were checked by NanoDrop and Urea PAGE. The gels were visualized using a laser scanner (Typhoon FLA 9000, GE Healthcare) with a 473 nm excitation laser and LPB filter after staining with SYBRGold for general detection of nucleic acids. Twenty pmol of the synthesized mRNA libraries were hybridized and photo-crosslinked to the cnvK linker (Fig. S5) on a 20-μL scale, as done before^19,34^. Six μL of the obtained reaction mixture (3.6–4.2 pmol of the mRNA-linker) was used as a template for in vitro translation using the PURE*frex* kit in a 25-μL scale, in triplicate. The reaction mixtures were incubated at 37 °C for 30 min, followed by the addition of EDTA (20 mM) and incubation at 37 °C for 5 min to release the ribosomes. In the following, the reactions were centrifuged, and supernatants containing mRNA display (mRNA-linker-protein) complexes were collected and first checked by Urea SDS-PAGE. The gels were visualized using a laser scanner with a 473 nm excitation laser and LPB filter or using a LED light imager (Biotools, Japan) equipped with a green band-pass filter for detection of mRNA-linker and mRNA display complexes via fluorescein attached to the cnvK linker. Purification was carried out by immobilization of mRNA display molecules from 60 μL of the total collected supernatant (4.3–5.0 pmol of mRNA display) to the streptavidin-coated magnetic microbeads (from 80 μL of the bead suspension) via biotin of the puromycin linker. After immobilization, the beads were washed three times with 200 μL of binding buffer (10 mM Tris–HCl, pH 8.0, 1 mM EDTA, 1 M NaCl, 0.1% Tween 20) and 1X ReverTra Ace buffer. Reverse transcription was then carried out on the beads to convert mRNA to cDNA using the ReverTra Ace (Toyobo) reverse transcriptase at 42 °C for 30 min on a rotator. Beads were then washed with 200 μL of selection buffer (50 mM Tris–HCl, pH 7.4, 0.5 M NaCl, 1 mM EDTA, 0.05% Tween 20), and treated with RNase T1 for 15 min at 37 °C on a rotator to release formed cDNA display (mRNA-cDNA-linker-protein) complexes by digestion of the RNase T1 recognition site included in the linker structure. Supernatant after RNase T1 treatment (40 μL) containing the cDNA display molecules was used for subsequent selection.

### Selection of transglutaminase substrate peptides

The selection was performed on a 100-μL scale, using 30 μL of cDNA display solution per reaction. In addition, each reaction mixture contained 32 mM pentylamine-biotin as acyl acceptor substrate, 10 mM Tris–HCl, pH 8.0, 15 mM CaCl_2_, 7.5 mM DTT and 0.01 mg/mL recombinant mouse TG2. The reaction was incubated at 37 °C for 90 min, followed by ultrafiltration with a 3 kDa cut-off membrane (Merck Millipore) to remove the unreacted pentylamine-biotin. In the following, formed covalent complexes between cDNA display molecules and pentylamine-biotin were pulled from the mixture by immobilization to streptavidin-coated magnetic microbeads (from 20 μL of the bead suspension). After immobilization, the beads were separated from the supernatant, washed three times with each, 1 mL of the selection buffer v4 (50 mM Tris–HCl, pH 7.4, 0.5 M NaCl, 1 mM EDTA, 0.7% Tween 20) and 100 μL of TE buffer, followed by resuspension in 15 μL of TE buffer.

### Analysis of the selected DNA

Analysis of the DNA selected from model libraries is described in the *Supplementary material* (Methods section). Original (before selection), enriched and leftover cDNA pools of the LibQ and Lib4 libraries were used for PCR amplification using the primers Nested_Fw1 and Nested_Rv1 (Table S2, Fig. S6) to check for the presence of target DNA bands in each of the fractions. For the enriched pools, different volumes of the PCR mixture after the first nested PCR were used as templates for the second nested PCR with Nested_Fw2 and Nested_Rv2 primers (Table S2, Fig. S5). Amplified DNA was analyzed by electrophoresis. DNA amplified from the original and enriched (S1 sample in Fig. S2) pools was purified and prepared for NGS analysis.

To prepare the amplified original and enriched DNA pools for sequencing, corresponding DNA (products after the second step of the nested PCR when 1 μL of the first PCR reaction was used as a template) was used for another PCR amplification with the following set of primers (Table S2): NGS prep (T26) Fw and Nested_Rv1 for Lib4 before and after selection, NGS prep (T26, randomQ, after) and Nested_Rv1 for LibQ after selection, and NGS prep (T26, randomQ, before) and Nested_Rv1 for LibQ before the selection. Since we analyzed the original and enriched LibQ DNA as a single sequencing sample, this PCR amplification step also introduced specific 10 bp tags upstream of the T26 gene to distinguish the sequence reads belonging to the original pool from the ones belonging to the enriched pool. After the PCR amplification, DNA was column-purified. Original and enriched LibQ DNA was mixed in a 1:1 molar ratio to make one sample for the analysis, while original and enriched Lib4 DNA were analyzed as separate two samples. The samples were further prepared and analyzed by paired-end next-generation sequencing (Illumina, NextSeq550) carried out at the Center for Gene Research of Nagoya University.

### Processing of the NGS data

At first, data quality was assessed by Seqkit^35^. Quality scores, Q20(%) and Q30(%), were between 93 and 95%, which indicates a low probability of incorrect base identification in our data sets. Trimmomatic^36^ was used to remove contaminating adapter sequences. Seqkit was applied for quality filtering at Q25. 82–83% of total sequences passed the evaluation and were further used. Reads from the sample containing the tagged sequences from LibQ were processed to separate the reads corresponding to the library before and after selection. The statistics of sequence files were monitored after each processing step to ensure sufficient data are retained for the next step. Finally, all bases upstream of the peptide sequence were removed from the reads and the remaining DNA sequence was converted into the amino acid sequence and analyzed by Biopython^37^. For Lib4, the final filtering of peptide sequences before and after the selection was applied to exclude sequences that do not contain Gln at the second position or have a stop codon somewhere in the peptide backbone, since those sequences are not planned according to the library design but could be present as a result of chemical synthesis of the DNA library and subsequent carryover. The rank list of top 100 enriched peptides further includes filtering of the sequences that show less than 100 counts after the selection, as these were not considered significant. The ranking was made based on the value of the enrichment factor (EF) (from highest to lowest). The sequencing counts obtained from the NGS were normalized to the total counts in each sample, and amino acid or peptide EF was expressed as the quotient of normalized counts before and after selection.

### Statistical data analysis

A Random Forest regression model was trained to predict the enrichment based on the one-hot encoded peptide sequences using the RandomForestRegressor module from scikit-learn v.0.24.2^38^. Model parameters were hypertuned by minimizing the mean squared error of enrichment predictions using Hyperopt v.0.2.5^39^. Hypertuning was performed for 1000 iterations, each using tenfold cross-validation and 200 estimators. The best performing model was re-trained using tenfold cross-validation and 1000 estimators, and for each cross-validation, the permutation feature importance was calculated ten times using the permutation_importance function from scikit-learn. The Pearson correlation coefficient for each feature was used to infer whether amino acids of high importance were associated with an increase or decrease of enrichment.

### TG2 enzymatic assay

The assay was done as described before^40^. 96-well immunoplate (Sumilon, Japan) was coated with 1 mg/mL β-casein in TBS for 30 min at 37 °C. After removal of the residual casein, the wells were blocked with wash buffer (TBS, 0.1% Tween 20) for 30 min at 37 °C, followed by washing with TBS buffer. 2 × peptide solution (40 mM TRIS–HCl pH 8.0, 280 mM NaCl, 30 mM CaCl_2_, 15 mM DTT, and varying concentrations of the biotinylated peptides in the range from 0 to 10 μM) was added at 50 μL/well, followed by 2 × TG2 solution (50 ng/mL of TG2 in TBS with 1 mM DTT and 1% BSA) at 50 μL/well. The reactions were incubated for 30 min at 37 °C. The quenching was done with 50 mM EDTA in TBS, and this was followed by washing with the wash buffer. The streptavidin-peroxidase solution in PBS containing 0.1% BSA and 0.1% Tween 20 was added at 100 μL/well. The reactions were incubated for 60 min at 37 °C. The wells were washed with wash buffer and TBS. TMB was added at 100 μL/well, followed by 1 N H_2_SO_4_ at 100 μL/well after the blue color appeared. Absorbance was measured at 450 nm after the development of the yellow color (30–60 s after the addition of H_2_SO_4_).

## Supplementary Information

Supplementary Information.

## Data Availability

Detailed information on the selected and tested (by TG2 enzymatic assay) peptide sequences are available at https://doi.org/10.6084/m9.figshare.20252976.v1. Raw data including all peptide sequences are to be shared upon request to the corresponding author.
