# Supplementary material for: Comprehensive analysis of transglutaminase substrate preference by cDNA display coupled with next-generation sequencing and bioinformatics
Source: Sci Rep. 2022 Aug 9;12:13578. doi: 10.1038/s41598-022-17494-4 (PMC9363462; doi:10.1038/s41598-022-17494-4)

**Supplementary material for:**

**Comprehensive analysis of transglutaminase substrate preference by cDNA display coupled with next-generation sequencing and bioinformatics**

**Jasmina Damnjanović**^1^***, Nana Odake**^1^**, Jicheng Fan**^1^**, Maurizio Camagna^2^, Beixi Jia**^1^**, Takaaki Kojima**^1^**, Naoto Nemoto**^3^**, Kiyotaka Hitomi**^4^**, Hideo Nakano**^1^

^1^Laboratory of Molecular Biotechnology, Graduate School of Bioagricultural Sciences, Nagoya University, Furo-cho, Chikusa-ku, Nagoya 464-8601, Japan

^2^Laboratory of Plant Genetics and Breeding, Graduate School of Bioagricultural Sciences, Nagoya University, Furo-cho, Chikusa-ku, Nagoya 464-8601, Japan

^3^Laboratory of Evolutionary Molecular Engineering, Graduate School of Science and Engineering, Saitama University, 255 Shimo-Okubo, Sakura-ku, Saitama 338-8570, Japan

^4^Laboratory of Cellular Biochemistry, Graduate School of Pharmaceutical Sciences, Nagoya University, Furo-cho, Chikusa-ku, Nagoya 464-8601, Japan

**Methods**

*Preparation of plasmids with T26 and T26A genes*

Plasmid pRSET-T26 with T26 peptide gene was prepared by inverse PCR with primers 1-2 listed in Table S2, using pRSET-T26-GST plasmid as a template. This was followed by ligation using Mighty Mix (Takara Bio).

Plasmid pRSET-T26A with the gene of non-substrate peptide, T26A with Q2A mutation, was obtained by inverse PCR with mutagenic primers 3-4 listed in Table S2 and pRSET-T26 as a template. In-fusion cloning kit (Takara Bio) was used to self-circularize the obtained DNA. Introduced Gln to Ala mutation resulted in the introduction of the *Sph*I restriction site.

*Preparation of DNA templates for cDNA display of binary model libraries*

Genes of T26 and T26A were amplified by PCR using the corresponding plasmids as templates, and New Left and cnvK_New Ytag primers listed in Table S2 (priming region shown in Fig. S3B). Amplified PCR products (Fig. S1A) were column-purified (QIAquick PCR Purification Kit, QIAGEN, Germany) and their concentration was evaluated by NanoDrop (NanoDrop, USA). Purified DNA was used for *in vitro* transcription.

*Analysis of DNA selected from model libraries*

Original cDNA library, beads suspension (enriched library) and supernatant fractions (leftover library) were used for PCR amplification of peptide DNA using the primers Nested_Fw1 and Nested_Rv1 (Table S2, Fig. S5). At the Q2A mutation site, T26A DNA contains a unique restriction site for *Sph*I, which T26 does not have. This property was used to distinguish between PCR-amplified T26 and T26A DNA. Briefly, 5 μL of PCR product was subjected to restriction digestion with *Sph*I and analyzed by agarose gel electrophoresis. The band pattern was compared with that of digested T26 and T26A original DNA to identify the enriched DNA.

**Figures and tables**

**
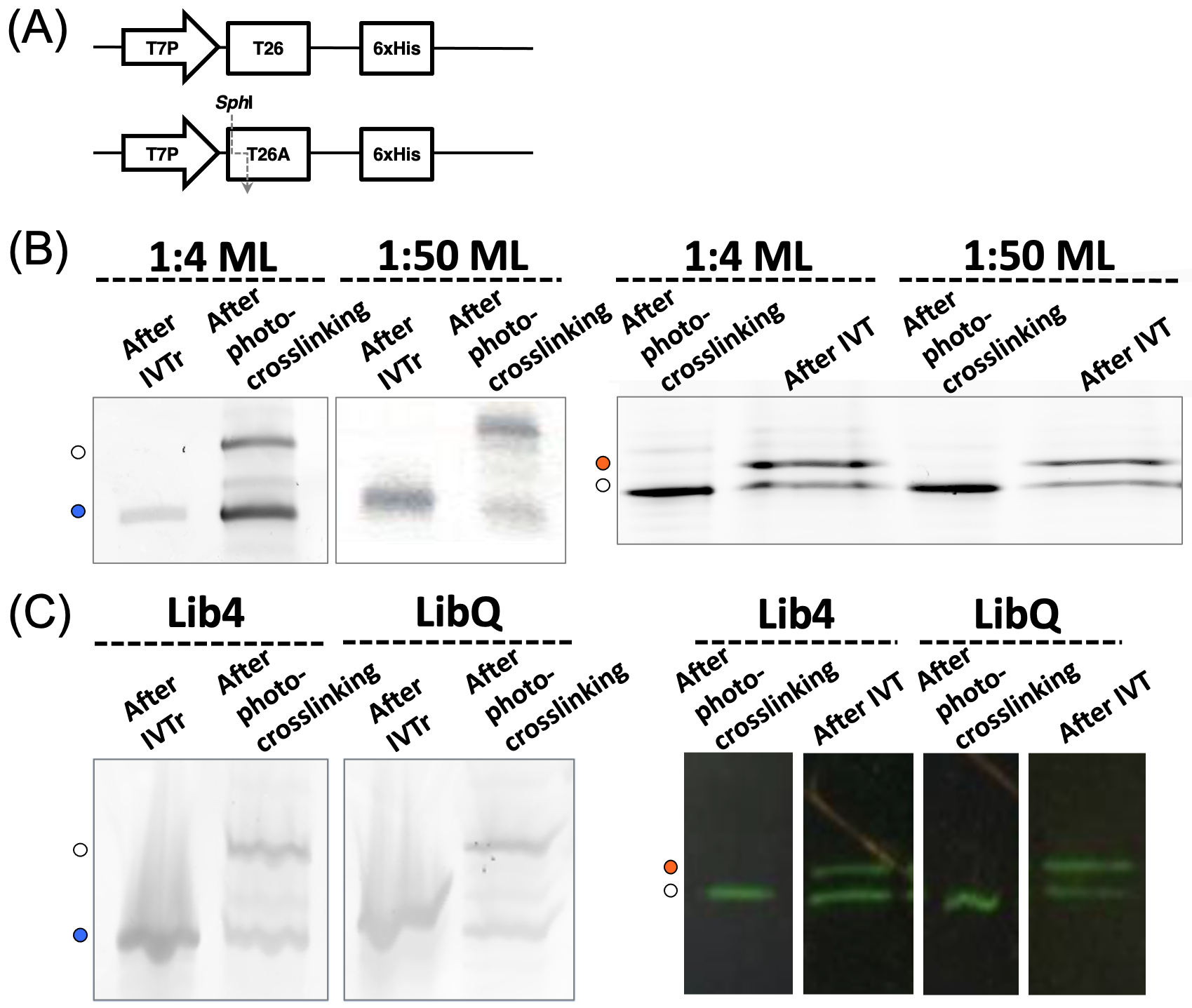
**

**Figure S1**. (A) Schematic representation of the T26 and T26A DNA templates used for cDNA display. (B) Analysis of the mRNA, mRNA-linker and mRNA displayed model libraries 1:4 and 1:50. Left panel: Urea PAGE of the purified mRNA libraries (after *in vitro* transcription (IVTr)) and corresponding crosslinking products detected by SYBRGold. Right panel: Urea SDS-PAGE of the crosslinking products and after *in vitro* translation (IVT) detected by fluorescence. (C) Analysis of the mRNA, mRNA-linker and mRNA displayed Lib4 and LibQ detected by SYBRGold. Left panel: Urea PAGE of the purified mRNA libraries (after *in vitro* transcription (IVTr)) and corresponding crosslinking products. Right panel: Urea SDS-PAGE of the crosslinking products and after *in vitro* translation (IVT) detected by fluorescence.

In left panels (B) and (C), blue circles indicate the position of the mRNA library and white circles indicate the position of the mRNA-linker library. In right panels (B) and (C), orange circles indicate the position of the mRNA display complex and white circles indicate the position of the mRNA-linker complex.


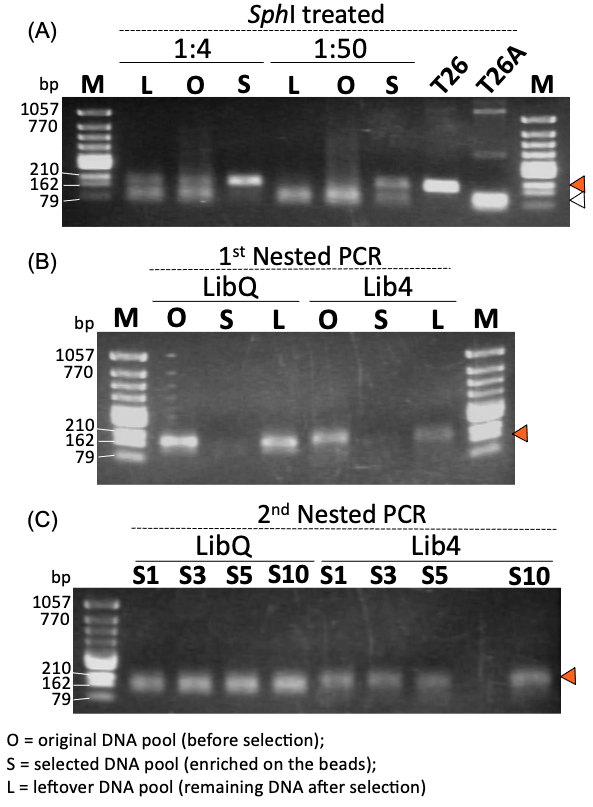


**Figure S2**. Post-selection analysis of the enriched DNA. (A) Agarose gel electrophoresis of PCR-amplified and *Sph*I-treated DNA from the original, selected, and leftover pools of the model libraries. (B) Agarose gel electrophoresis after PCR amplification (first step of the nested PCR) of DNA from the original, selected, and leftover pools of the random libraries. (C) Electrophoresis after PCR amplification (second step of the nested PCR) of the selected DNA from random libraries. Lanes S1, S3, S5 and S10 show the bands of the PCR products after the second step of the nested PCR when 1, 3, 5, and 10 μL of the first PCR reaction was used as a template respectively. The orange triangle indicates the position of amplified DNA bands.


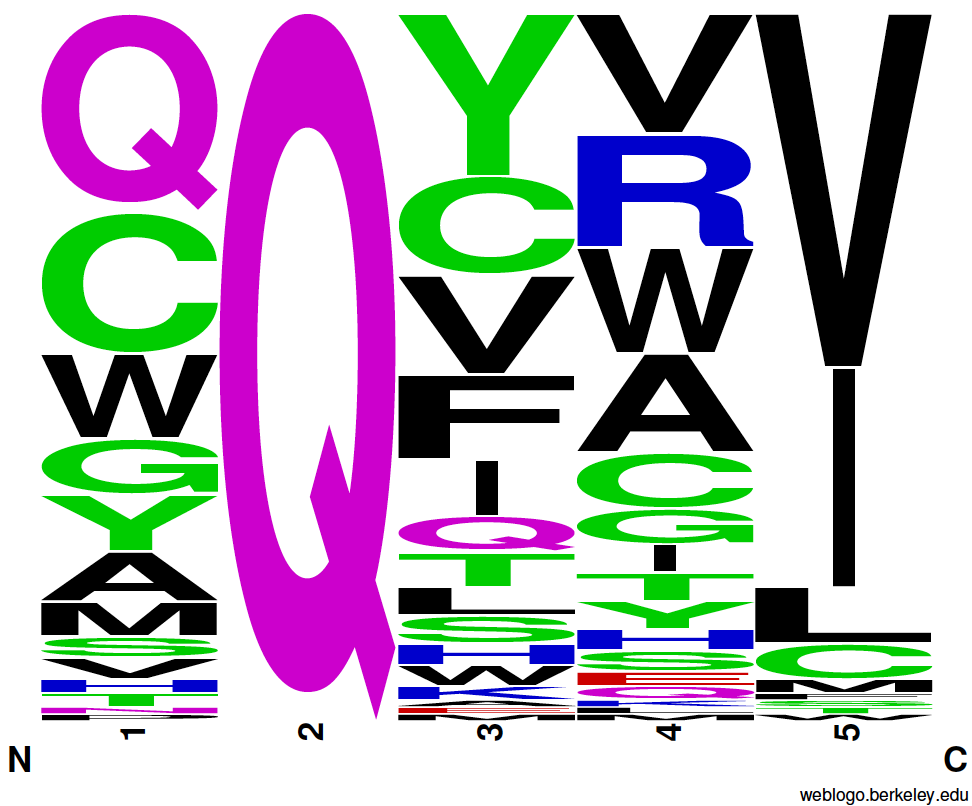


**Figure S3**. Sequence logo of the top 100 enriched peptides from the Lib4 generated by WebLogo (https://weblogo.berkeley.edu/logo.cgi).


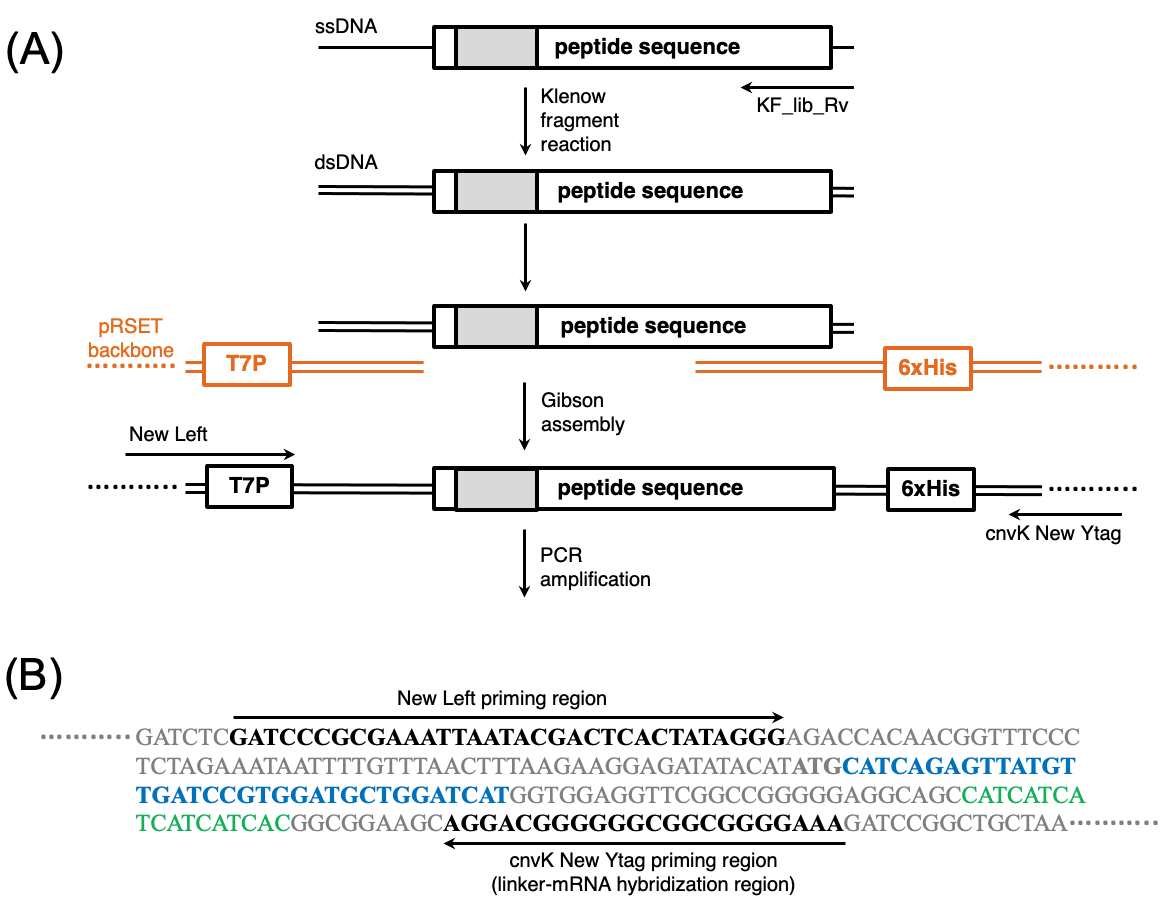


**Figure S4**. (A) Preparation of the DNA template of the random libraries, LibQ and Lib4. Gray color marks the part of the peptide with randomized sequence. (B) Priming regions of the New Left and cnvK_New Ytag primers. Blue color indicates the peptide sequence, while green indicates sequence of the 6xHis tag.


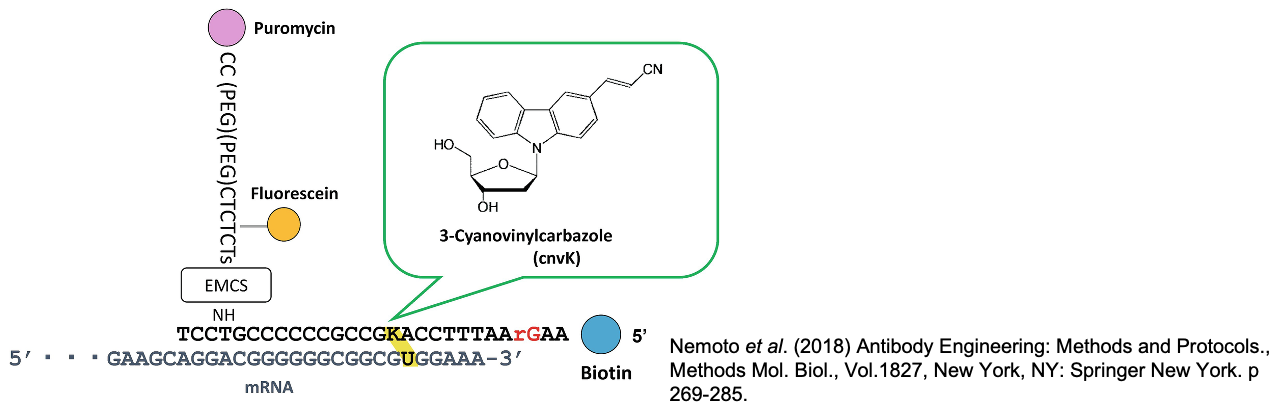


**Figure S5.** Structure of the puromycin cnvK linker used in this study. RNase T1 restriction site is indicated in red (rG).


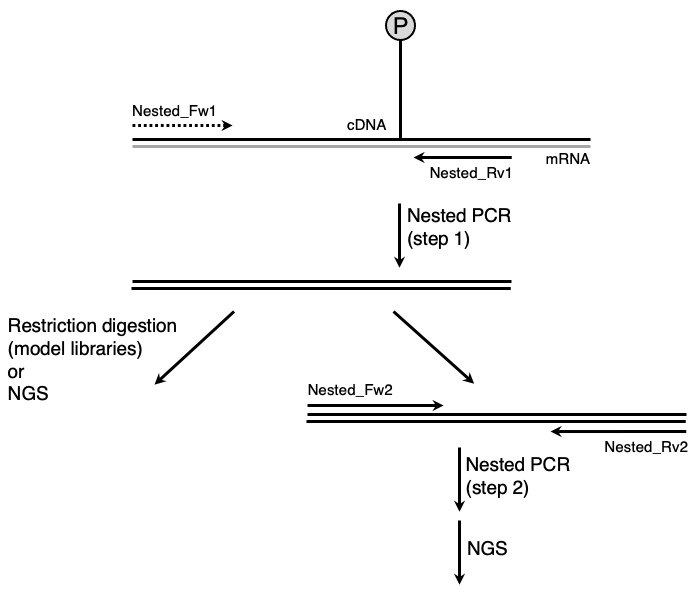


**Figure S6**. Post-selection preparation of the enriched DNA for analysis. The first step involves PCR, after which DNA is either used for analysis by restriction digestion (model libraries) or NGS (original LibQ and Lib4, before the selection), or for the second PCR (enriched complexes from LibQ and Lib4, after the selection) followed by NGS.

**Table S1.** Rank list showing the top 100 peptide sequences enriched from Lib4.

| Ranking | Peptide sequence  (*X*Q*XXX*DPWMLDH)  * only first 7 a.a. shown | Enrichment factor |
| --- | --- | --- |
| 1 (Top 1) | QQVCIDP | 7.12 |
| 2 (Top 2) | QQYVVDP | 6.62 |
| 3 | QQFVVDP | 6.17 |
| 4 | QQFRVDP | 5.72 |
| 5 | QQVWVDP | 5.35 |
| 6 | QQYRVDP | 5.31 |
| 7 | WQTWVDP | 5.22 |
| 8 | PQVWFDP | 5.10 |
| 9 | AQWYVDP | 5.10 |
| 10 | CQFAMDP | 5.06 |
| 11 | HQFAVDP | 4.96 |
| 12 | MQYAVDP | 4.94 |
| 13 | CQTWIDP | 4.92 |
| 14 | SQCWIMV | 4.90 |
| 15 | QQYLVDP | 4.90 |
| 16 | QQYGLDP | 4.87 |
| 17 | MQFAVDP | 4.86 |
| 18 | WQHVVDP | 4.48 |
| 19 | CQKCVDP | 4.47 |
| 20 | QQIVVDP | 4.46 |
| 21 | AQCAIDP | 4.42 |
| 22 | QQVICDP | 4.33 |
| 23 | QQCRVDP | 4.29 |
| 24 | QQVILDP | 4.22 |
| 25 | CQYRIDP | 4.21 |
| 26 | QQSSIDP | 4.20 |
| 27 | CQCAIDP | 4.17 |
| 28 | CQTWVDP | 4.17 |
| 29 | CQYWVDP | 4.17 |
| 30 | QQIGVDP | 4.14 |
| 31 | WQCRIDP | 4.14 |
| 32 | YQFEVDP | 4.10 |
| 33 | QQVIVDP | 4.07 |
| 34 | AQYWVDP | 4.03 |
| 35 | SQQCVDP | 4.01 |
| 36 | YQYRCDP | 4.01 |
| 37 | YQCWIMV | 3.99 |
| 38 | WQCHVDP | 3.97 |
| 39 | WQSIIDP | 3.95 |
| 40 | CQYALDP | 3.95 |
| 41 | QQWVLDH | 3.95 |
| 42 | GQKYVDP | 3.94 |
| 43 | GQCWIMV | 3.93 |
| 44 | TQCAVDP | 3.93 |
| 45 | WQYRIDP | 3.93 |
| 46 | YQITVDP | 3.91 |
| 47 | AQHRVDP | 3.91 |
| 48 | AQEWVDP | 3.88 |
| 49 | GQQVIDP | 3.88 |
| 50 | WQCEVDP | 3.87 |
| 51 | QQVYVDP | 3.84 |
| 52 | QQYRTDP | 3.81 |
| 53 | QQSWVDP | 3.81 |
| 54 | VQQVIDP | 3.81 |
| 55 | MQVWIDP | 3.79 |
| 56 | WQFTVDP | 3.78 |
| 57 | WQCVIDP | 3.78 |
| 58 | QQVRCDP | 3.78 |
| 59 | AQYVVDP | 3.76 |
| 60 | QQIVLDH | 3.75 |
| 61 | QQVRIDP | 3.75 |
| 62 | WQTVCDP | 3.75 |
| 63 | QQSRVDP | 3.73 |
| 64 | CQVWVDP | 3.71 |
| 65 | CQYTIDP | 3.71 |
| 66 | MQFGIDP | 3.71 |
| 67 | GQLCWIM | 3.70 |
| 68 | YQYCVDP | 3.69 |
| 69 | CQYAMDP | 3.68 |
| 70 | QQYVSDP | 3.68 |
| 71 | YQLCVDP | 3.66 |
| 72 | CQQSVDP | 3.65 |
| 73 | QQVRVDP | 3.65 |
| 74 | GQFTVDP | 3.63 |
| 75 | YQVHIDP | 3.63 |
| 76 | WQVQLDP | 3.62 |
| 77 | SQQYVDP | 3.62 |
| 78 | AQFAVDP | 3.60 |
| 79 | QQLWIDP | 3.60 |
| 80 | CQIVCDP | 3.60 |
| 81 | QQIGLDP | 3.59 |
| 82 | TQYAVDP | 3.59 |
| 83 | GQYQVDP | 3.59 |
| 84 | CQCVIDP | 3.59 |
| 85 | CQTRIDP | 3.57 |
| 86 | GQHVIDP | 3.57 |
| 87 | GQCMIDP | 3.56 |
| 88 | MQFVVDP | 3.55 |
| 89 | CQLGIDP | 3.54 |
| 90 | VQYCIDP | 3.53 |
| 91 | CQMCIDP | 3.53 |
| 92 | CQIRIDP | 3.52 |
| 93 | VQCAIDP | 3.52 |
| 94 | CQYHVDP | 3.51 |
| 95 | QQYVLDP | 3.51 |
| 96 | HQYAVDP | 3.51 |
| 97 | WQIRVDP | 3.50 |
| 98 | NQFAVDP | 3.50 |
| 99 | YQAKVDP | 3.49 |
| 100 | CQWSIDP | 3.48 |

* N-terminal Met is present in all peptides and represents a translated start codon which remained uncleaved due to the absence of methionine aminopeptidase in the PURE system. It is omitted from the peptide sequences in the list since it does not represent preference of TG2.

**Table S2.** List of oligonucleotide primers used in this study.

| Number | Name | Sequence (5'-3') | Use |
| --- | --- | --- | --- |
| 1 | T26 only_Fw | GGGGGAGGCAGCCATCATCATCATC | Preparation of pRSET-T26 |
| 2 | T26 tag (vector)_Rv | GGCCGAACCTCCACCATGATCCAG |  |
| 3 | T26_QtoA_Fw | GCGAGTTATGTTGATCCGTGGATGCTG | Preparation of pRSET-T26A |
| 4 | T26_QtoA_Rv | ATCAACATAACTCGCATGCATATGTATATCTCC |  |
| 5 | New Left | GATCCCGCGAAATTAATACGACTCACTATAGGG | Preparation of DNA template for cDNA display |
| 6 | cnvK_New Ytag | TTTCCACGCCGCCCCCCGTCCT |  |
| 7 | KF_lib_Rv | CCATGATCCAGCA | Klenow fragment reaction |
| 8 | GA_vec_lib_Fw | GTGGATGCTGGATCATGGTGGA | Preparation of pRSET for Gibson assembly to dsDNA library |
| 9 | GA_vec_lib_Rv | TGTATATCTCCTTCTTAAAGTTAAACAAAATTAT |  |
| 10 | Nested_Fw1 | GGGAGACCACAACGGTTTCC | Amplification of DNA after selection |
| 11 | Nested_Rv1 | TTTCCCCGCCGCCCCC |  |
| 12 | Nested_Fw2 | GGGAGACCACAACGGTTTCCCTCTAGAAAT |  |
| 13 | Nested_Rv2 | TTTCCCCGCCGCCCCCCGTC |  |
| 14 | NGS prep (T26)_Fw | CCCTCTAGAAATAATTTTGTTTAACTTTAAG | Preparation of libraries for NGS |
| 15 | NGS prep (T26, randomQ, after) | CTGACAAAAACCCTCTAGAAATAATTTTGTTTAACTTTAAG |  |
| 16 | NGS prep (T26, randomQ, before) | CTGACTTTTTCCCTCTAGAAATAATTTTGTTTAACTTTAAG |  |

Appendix. Full-size gel images of data presented in Figures S1B and C, and S2.

1.
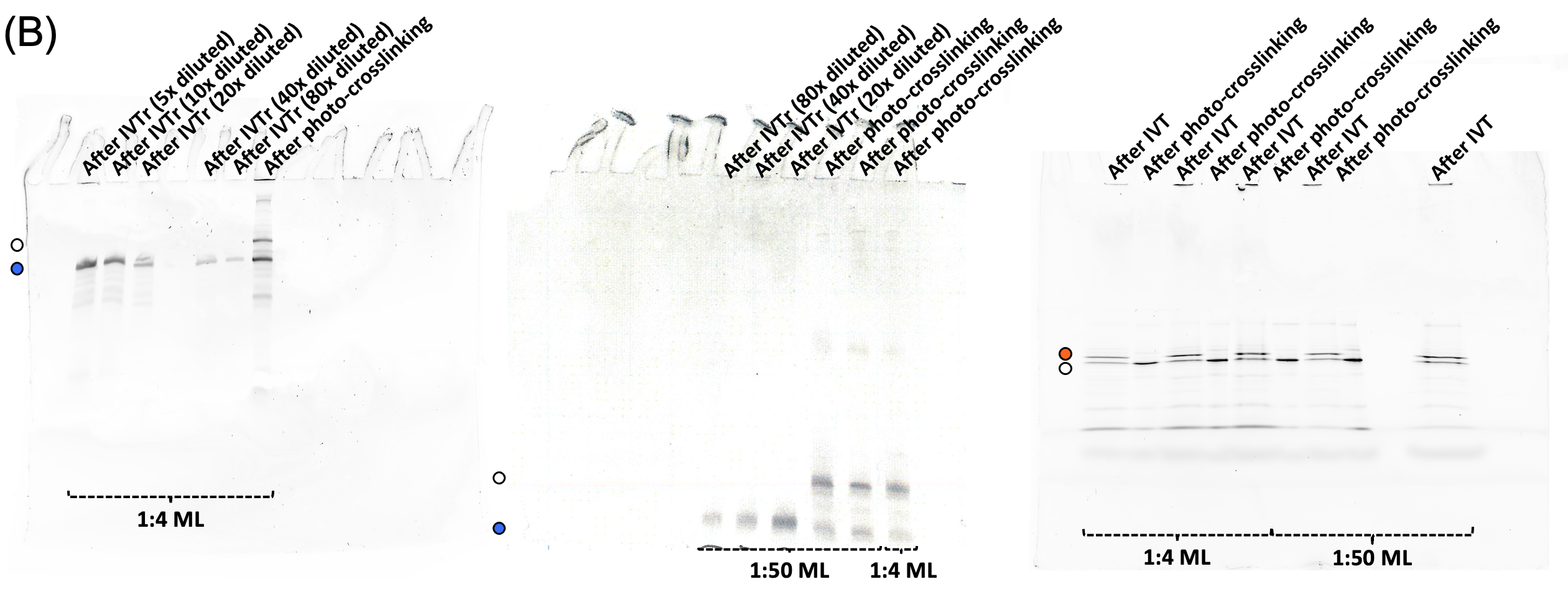
Full-size gel image of data presented in Figure S1B and C.


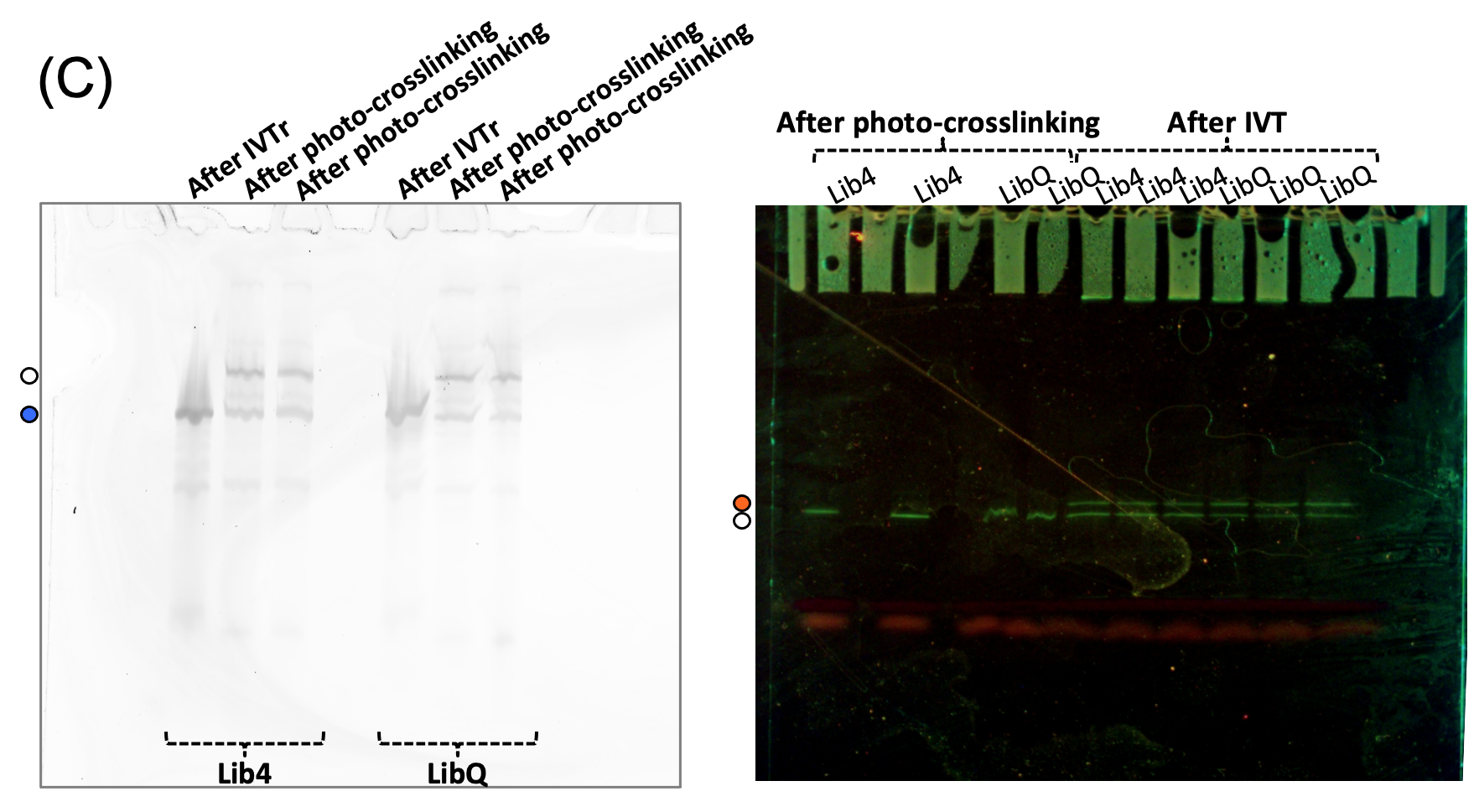


1. Full-size gel images of data presented in Figure S2.


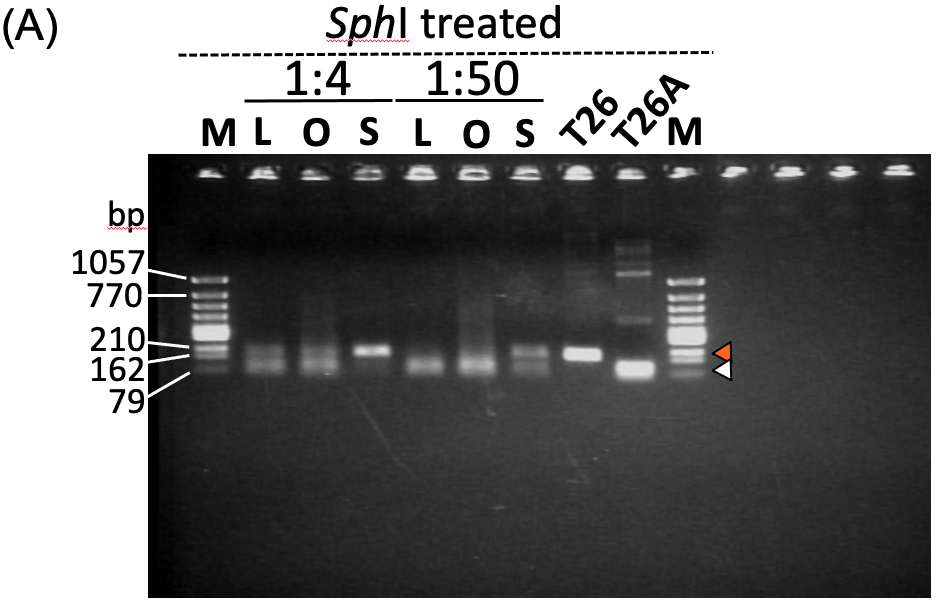


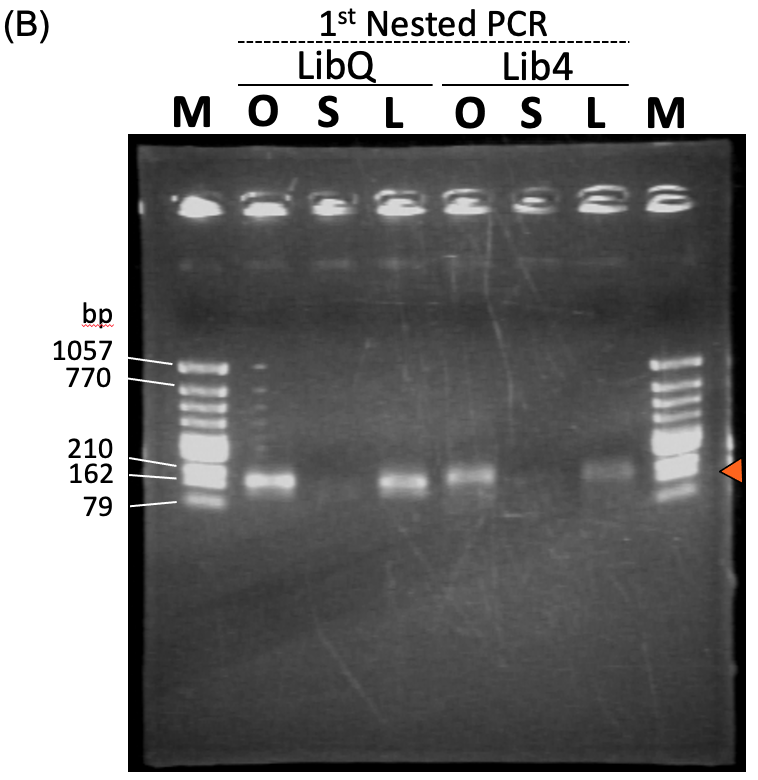


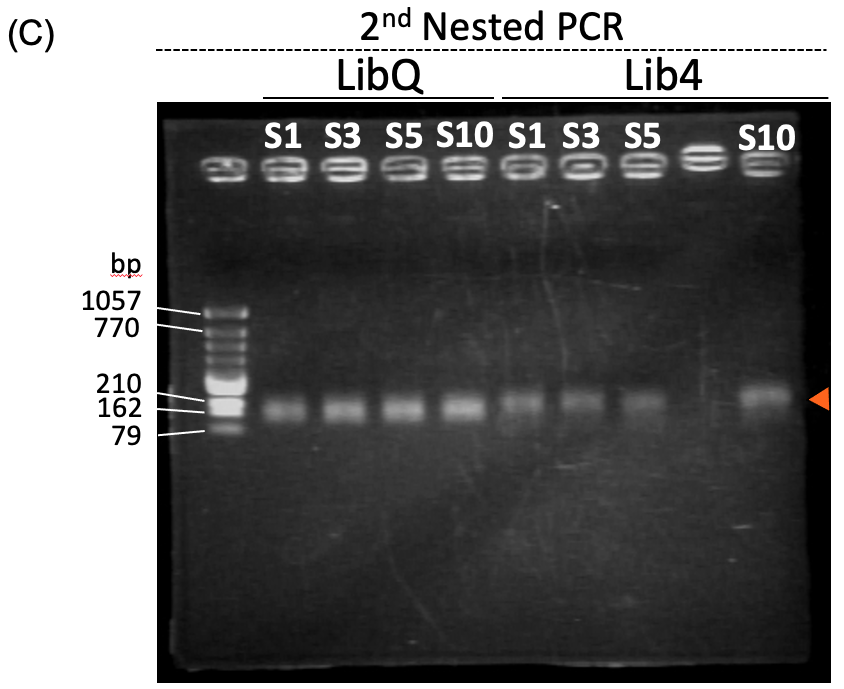

Supplement: Supplementary file 1 — Supplementary Information. [file 41598_2022_17494_MOESM1_ESM.docx]
